# The influence of genetic and acquired factors on the vulnerability to develop depression: a review

**DOI:** 10.1042/BSR20222644

**Published:** 2023-05-23

**Authors:** Xingfang Zhang, Yajun Qiao, Mengyuan Wang, Xinxin Liang, Ming Zhang, Cen Li, Jixian Cairang, Jianv Wang, Hongtao Bi, Tingting Gao

**Affiliations:** 1 School of Psychology, Chengdu Medical College, Chengdu 610500, China; 2 Qinghai Provincial Key Laboratory of Tibetan Medicine Pharmacology and Safety Evaluation, Northwest Institute of Plateau Biology, Chinese Academy of Science, Xining 810008, China; 3 Medical College, Qinghai University, Xining 810001, China; 4 Department of Psychiatry, the People’s Hospital of Jiangmen, Southern Medical University, Jiangmen 529000, China; 5 CAS Key Laboratory of Tibetan Medicine Research, Northwest Institute of Plateau Biology, Chinese Academy of Sciences, Xining 810001, China; 6 Department of Cardiovascular and Cerebrovascular, Tibetan Medicine Hospital of Qinghai Province, Xining 810007, China

**Keywords:** acquired factors, congenital factors, Depression, mental health

## Abstract

Depression is one of the most common mental disorders that affects hundreds of millions of people worldwide and has claimed tens of thousands of lives. The causes are divided into two main areas: congenital genetic factors and acquired environmental factors. Congenital factors include genetic mutations and epigenetic events; acquired factors include birth patterns, feeding patterns, dietary patterns, childhood experiences, education and economic levels, isolation due to epidemics, and many other complex factors. According to studies, these factors play important roles in depression. Therefore, here, we analyze and study the factors from two aspects, describe their influence on individual depression, and analyze their underlying mechanisms. The results showed that both innate and acquired factors have significant effects on the occurrence of depressive disorder, and these findings may provide new ideas and methods for the study of depressive disorder, thus facilitating the process of depression prevention and treatment.

## Introduction

In recent years, depression as a common neurological disorder has gradually attracted widespread social attention, especially during the recently passed novel coronavirus pneumonia epidemic, where the incidence of depression increased dramatically, with the World Health Organization reporting a 25% increase in the prevalence of depression and anxiety disorders during the epidemic [1]. Depression is characterized by persistent sadness, and depending on the condition, mood can exhibit varying degrees of abnormality [2]. Common clinical manifestations of depression include depressed mood, impaired thinking, reduced volitional activity, impaired cognitive function, and somatic symptoms such as loss of vitality, insomnia or drowsiness, weight loss or weight gain, mental stimulation, easy fatigue, and suicidality [3]. It is well known that the presence of suicidal tendencies in patients is related to the severity of the illness, and patients with severe illness will feel extreme pain, feel that life is meaningless, develop feelings of pessimism and anxiety, and choose to relieve their pain through suicidal thoughts. Several studies have analyzed the factors associated with suicide risk in depression, such as the study by Conejero et al. [4], in which psychological pain was found to be a prominent aspect of depression and was associated with a higher risk of suicidal thoughts and suicidal behavior. Rogers et al. noted that depression-related somnolence may be a protective factor against suicide attempts, while increased arousal may be a suicide risk factor [5]. Depression is a serious threat to human physical and mental health, and it has become a serious medical condition and a major public health problem. Therefore, research on depression, including influencing factors [6], pathogenesis [7], and drug research [8], has attracted extensive interest from researchers.

Currently, there are several hypotheses for the development of depression: (1) The first proposed monoamine neurotransmitter abnormality hypothesis suggests that depression occurs due to decreased levels of monoamine neurotransmitters such as 5-hydroxytryptamine and norepinephrine in patients [9]. (2) The oxidative stress dysfunction hypothesis, which is based on the discovery of a disrupted antioxidant system in depressed patients and abnormal levels of oxidative products [10]. (3) The cytokine abnormality hypothesis, a growing number of studies have shown that there is an interconnection between immune, endocrine and neurotransmitters, and a severe imbalance of inflammatory factors has been found in depressed patients, which play an important role in behavioral, neurological, and endocrine regulation [11,12]. (4) The HPA axis abnormality hypothesis suggests that stressful stimuli lead to HPA axis hyperactivity, which in turn leads to abnormal levels of CRH, ACTH, and CORT in the body fluids of depressed patients [13]. (5) The gut–brain axis hypothesis related to gut flora suggests that there is bidirectional communication between the gut flora and the brain and that metabolites of the flora may influence the inflammatory response and brain activity [14,15]. Indeed, a variety of neurotransmitter, immune system, and gut flora levels are abnormal in depressed patients; however, no clear conclusions have been made about the initial causes of these abnormalities in the organism.

Synthesizing the recent literature, the initiating factors that trigger depression can be grouped into two main categories. The first group of factors is due to congenital inheritance, mainly due to the genetic relationship of the parental generation, which includes genetic mutations, polymorphisms of genes and epigenetic events. The second group of factors is due to the influence of the individual’s acquired environment, which includes mainly birth and feeding patterns, dietary habits, childhood experiences, economic and educational level, and spatial constraints. All these factors play significant roles in the development and progression of depression. Therefore, to deepen the study of depression and to investigate the underlying factors that influence the development of depression, we investigated the relationship between depression and ‘epigenetic’ and ‘genetic mutations’. We examined the relationship between depression and ‘mode of birth’, ‘genetic mutation’, ‘birth mode’, ‘feeding pattern’, ‘diet’, ‘childhood experience’, ‘isolation’, ‘economic level’, and ‘education level’, and the literature was collected in PubMed and Google Scholar platforms. Endnote software was used to organize the literature to conduct the retrospective study of this paper.

**Figure 1:**
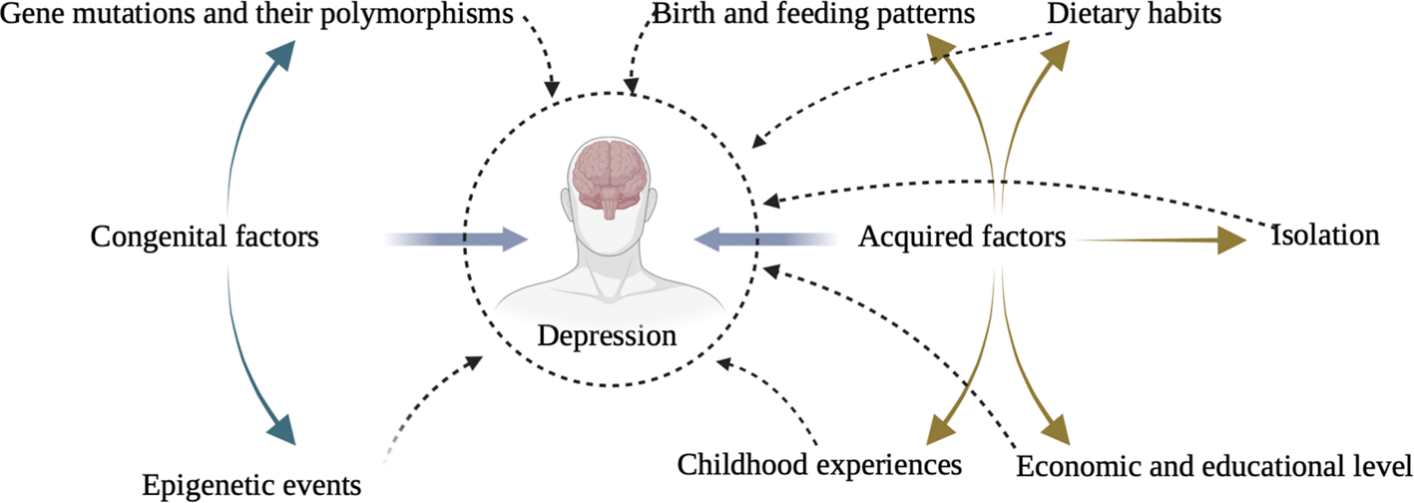
Factors influencing the onset and progression of depression

This paper explains the possible mechanisms by developing a discussion of innate genetic and acquired environmental factors and by combining them with physiological and biochemical changes in the organism. The aim of this study is to provide new ideas and perspectives to facilitate the research process of depression in clinical settings. The specific idea is shown in Figure 1.

## Congenital factors

Current research suggests that depression may be passed on to offspring through the parental generation. Discoveries and advances have also been made in the genetics of depression through genome-wide association analysis studies [16]. For example, it has been reported that perinatal anxiety and depression in pregnant women can affect the mental state of their offspring and can even lead to depression in children [17]. In a between-family analysis, Singh et al. showed a significant positive association between parental depression and offspring depression and behavioral disorders, which may be due to genetic factors [18]. Women seem to play a focal role in depression, which may be inextricably linked to female-specific biological factors, as progesterone and estrogen can influence neurotransmitter transmission and neuroendocrine function, which can directly affect a woman’s mood. Numerous studies have shown that depression in women during pregnancy has a great impact on offspring [19]. Both Eshim and Harold's studies have shown a direct link between maternal depression and depressive disorder in children [20,21]. The various studies above demonstrate the importance of genetic factors in depression.

### Gene mutations and their polymorphisms

Gene mutations and genetic polymorphisms play vital roles in many diseases, such as anxiety [22], depressive disorder [23], and Alzheimer’s disease[24]. Genetic studies on depression have conducted in-depth studies on genetic mutations and genetic polymorphisms. To date, more than 100 genetic risk loci have been identified [25].

Studies in heterozygous mice for the VMAT2 gene have shown that mutant animals exhibit many features of depression, such as a lack of pleasure, motor retardation, and sensitivity to stress, which may be explained by the fact that VMAT2 can have a controlling effect on the secretion of DA, NE, and 5-HT at the synapse [26]. Mutations in the 124 bp allele of D2S2944 are strongly associated with major depressive disorder (MDD) [27,28]. The association of D2S2944 with depression was found to be specific and appears to be sex-specific by comparing major depressive relapseers, nonpsychotic patients and early-onset individuals, possibly due to a chain imbalance of susceptibility genes in close proximity to the marker loci [28,29]. A meta-analysis showed that a homozygote mutation in NR3C1 rs41423247 was associated with depression in the total population (OR = 0.77, 95% CI = 0.64–0.94, *P*=0.01) and in Caucasians (OR = 0.78, 95% CI = 0.63–0.96, *P*=0.02) [30], and Cuzzoni et al. found that the NR3C1 rs41423247 (C/G) polymorphism may influence the onset of depression by altering the function of the glucocorticoid receptor [31]. When gender differences in the genetic structure of depression were investigated, five genetic markers of increased risk of depression in women were obtained by qualitative analysis, three of which were rs201432982 in PDE4A, rs62640397, and rs79442975 in FDX1L, and the remaining two were rs820182 and rs820148 in MYO15B [32]. Variants of PDE4A may reduce neuronal firing and dysregulate negative feedback through the hypothalamic‒pituitary‒adrenal axis, leading to the development of depressive disorders [32]. Variants of FDX1L are associated with mitochondrial dysfunction and may promote the development of depression by causing oxidative stress and neurotransmitter release, thus increasing stress hormone levels, and are sex-specific [33–35]. Chromosome 17q25.1, mapped by MYO15B, is associated with an increased risk of cognitive dysfunction, dementia, and depression. Gene polymorphism studies have shown that the interaction of the 5-HTTLPR and COMT hypoactive alleles may be associated with reduced interconnectivity within emotional processing circuits, including frontal, temporal, and occipital regions and the right amygdala, which increases the risk of developing MDD [36]; a combination of 5-HT1A GG and BDNF GA + Aa genotypes is associated with a significantly increased risk of depression [37]; interleukin (IL)-10 family cytokines (IL-10, IL-19, IL-20, and IL-24) are all involved in inflammatory processes, and an increased risk of MDD associated with IL-20 and IL-24 haplotypes was identified by the study [38]; genetic variants in the IL-6 gene increased the risk of IFN-induced depression [39]; and mice with an overall deletion of the GABRA3 gene have more depression-related behaviors, which may be related to the unique position of the α3-GABAA receptor in regulating monoaminergic function [40].

Based on the above description, it is suggested that genetic variants at genetic risk loci in depressed parents may be passed on to their offspring, leading to the development of depression in the offspring.

### Epigenetic events

Over the past 60 years, the definition of ‘epigenetics’ has been continuously updated as the molecular mechanisms of eukaryotic gene expression regulation have been studied, with the main focus of this discipline being ‘the study of inheritable changes in gene function that cannot be explained by DNA sequence changes during mitosis and meiosis’. As depression has been studied more closely, epigenetic modifications, such as DNA methylation and histone modifications, have been identified to alter chromatin structure, thereby regulating the expression of genes that play a role in neuronal plasticity, behavioral responses to stress, depressive behavior, and responses to antidepressants.

DNA methylation normally reduces gene expression, and DNA methyltransferases (DNMTs), such as DNMT1, DNMT2, DNMT3A, DNMT3B, and DNMT3L, can catalyze DNA methylation; DNMT1 and DNMT3A are expressed at high levels in the somatic nuclei of mice exposed to major depression-like behaviors, which may regulate synaptic and structural plasticity as well as memory formation [41]. A review mentioned that DNA methylation modifications are associated with depression, with hypermethylation at sites encoding brain-derived neurotrophic factor (BDNF) and SLC6A4 (5-hydroxytryptamine transporter gene) [42]. The BDNF gene promotes the survival of nerve cells (neurons) and is actively involved in the growth, maturation and maintenance of these neurons, as well as in the regulation of synaptic plasticity [43,44], and methylation of BDNF leads to impaired neuronal plasticity, which may mediate the development of depression [45]. SLC6A4 provides instructions to make a protein in the brain that is involved in regulating 5-hydroxytryptamine signaling by transporting 5-hydroxytryptamine from the synaptic space to presynaptic neurons, and DNA hypomethylation may lead to a decrease in SLC6A4 expression and 5-HT reuptake, which in turn may increase vulnerability to affective disorders at critical stages of development [46,47]. Whole methylome association analysis revealed that the relevant differentially methylated regions in the blood of patients with major depression overlapped significantly with differentially methylated regions in the Brodmann area of the brain, and three CpG pooling sites in the brain were obtained: GABBR2, RUFY3, and the intergenic region on chromosome 2 [48]. GABBR2 inhibits neuronal activity through a G-protein-coupled second messenger system, while RUFY3 is associated with the establishment of neuronal polarity and axon elongation, loci involved in biological functions that may be important for the etiology of MDD.

Acetylation and deacetylation, as covalent histone modifications, affect chromatin structure and therefore regulate genes. In a study of histone modifications in depressed patients, it was found that global acetylation of histone 3 at lysine 14 (H3K14ac) was increased in the nucleus accumbens and therefore HDAC2 was downregulated [49]. Furthermore, it was found that chronic stress enhanced HDAC2 function and inhibited glial cell-derived neurotrophic factor (GDNF) transcription, thereby inducing depression-like behavior, and that aberrant regulation of HDAC4/5 expression and subsequent aberrant acetylation of histones led to depression [41]. Studies of histone modifications in the prefrontal cortex of postmortem MDD patients have revealed that the SYN1 promoter is enriched for trimethylated H3K4, a modification that is commonly associated with transcriptional activity [50]. Postmortem studies of MDD patients who were treated with antidepressants during life revealed that aberrant H3K4me3 or H3K27me3 was also found in the promoter regions of OAZ1, TRKB, and BDNF in the prefrontal cortex [51–53]. Another review study showed that epigenetic changes in NRC31, SLCA4, BDNF, FKBP5, SKA2, OXTR, LINGO3, POU3F1, and ITGB1, all stress-related genes, were associated with depression, where glucocorticoid signaling (e.g., NR3C1 and FKBP5), serotonergic signaling (e.g., SLC6A4) and neuro epigenetic changes in dystrophin (e.g., BDNF) genes appear to be the most promising therapeutic targets for future research [54].

The above-mentioned information on the influence of congenital factors on the onset and development of depression has been compiled and is shown in Table 1.

**Table 1:** List of congenital factors mediating the development of depression

Congenital factors	Point of view	Source
Gene mutations and their polymorphisms	VMAT2 mutant animals exhibit depressive symptoms such as lack of enjoyment, motor retardation and sensitivity to stress.	[26]
	Mutations in the 124 bp allele of D2S2944 are closely associated with MDD and appear to be sex specific.	[27–29]
	Homozygous mutations in NR3C1 rs41423247 are associated with depression.	[30]
	The study identified five genetic markers for increased risk of depression in women, three of which were rs201432982 for PDE4A, rs62640397, and rs79442975 for FDX1L, and the remaining two were rs820182 and rs820148 for MYO15B.	[32]
	Polymorphisms in 5-HTTLPR and COMT are associated with susceptibility to MDD development.	[36]
	The combination of 5-HT1A GG and BDNF GA + Aa genotypes is associated with a significantly increased risk of depression.	[37]
	Increased risk of MDD associated with IL-20 and IL-24 haplotypes.	[38]
	Genetic variants in the IL-6 gene increase the risk of IFN-induced depression.	[39]
	Mice with overall deletion of the GABRA3 gene have more depression-related behaviors.	[40]
Epigenetic events	DNMT1 and DNMT3A are highly expressed in the somatic nuclei of mice exposed to major depressive-like behaviors.	[41]
	DNA methylation modifications are associated with depression, with hypermethylation at sites encoding BDNF and SLC6A4.	[42]
	Relevant differentially methylated regions in the blood of patients with major depression significantly overlap with differentially methylated regions in the Brodmann area of the brain and yield three CpG pooling sites in the brain: GABBR2, RUFY3 and an intergenic region on chromosome 2.	[48]
	Chronic stress enhances the function of HDAC2, which inhibits the transcription of glial cell-derived neurotrophic factor (GDNF) and thus induces depression; abnormal expression of HDAC4/5 and subsequent abnormal acetylation of histones leads to depression.	[41]
	Studies of histone modifications in the prefrontal cortex of postmortem MDD patients showed that the SYN1 promoter is enriched for trimethylated H3K4.	[50]
	Autopsy results of MDD patients who had been treated with antidepressants showed that abnormal H3K4me3 or H3K27me3 was found in the promoter regions of OAZ1, TRKB, and BDNF in the prefrontal cortex.	[51–53]
	Epigenetic changes in NRC31, SLCA4, BDNF, FKBP5, SKA2, OXTR, LINGO3, POU3F1, and ITGB1, all stress-related genes, have been associated with depression.	[54]

## Acquired factors

Acquired environmental factors are extremely important for the development of an individual throughout his or her life. According to the relevant literature, the family environment and social environment influence the formation of a person’s personality and play a crucial role [55,56]. There are many acquired factors that we experience between birth and death, including our birth and feeding patterns, dietary habits, childhood experiences, educational and economic level, and the current global novel coronavirus epidemic we are experiencing. These factors have a significant impact on the occurrence and development of depression. Epidemiological studies have shown that stressful life events are associated with a high risk of MDD [57].

### Birth and feeding patterns influence depression through gut flora

The mode of birth was divided into normal delivery and cesarean section, and the mode of feeding was divided into breastfeeding and artificial feeding. Several studies have reported that differences in the birth and feeding methods of infants have different effects on children’s mental states and behavioral patterns [58], and the development of mental disorders such as depression in adulthood is one of their effects, in which intestinal flora plays an important role [59,60].

Studies have shown that birth and feeding practices influence the type and abundance of the infant’s gut flora [61,62]. It has been reported that when an infant is delivered by normal birth, the composition of the infant’s flora is similar to that of the mother’s vagina, which is beneficial to the infant, and when the infant is delivered by cesarean section, the composition of the infant's flora is similar to that of the flora on the mother’s skin, which is detrimental to the infant [63], meanwhile, common colonizing flora in infants born via cesarean sections are *Enterococcus*, *Enterobacter*, and *Klebsiella* species [64]. These pathogenic bacteria usually have an adverse effect on the organism. In terms of feeding practices, individuals who were breastfed have a higher proportion of beneficial bacteria in their intestinal flora and are less likely to develop mental illness than those who were not breastfed [65]. Studies have reported that beneficial bacterial genera such as *Bifidobacterium*, *Bacteroides*, *Clostridium*, and *Lactobacillus* dominate in breastfed infants [66,67]. Montgomery’s study showed that breastfeeding facilitates resistance to social stress [68]. Similarly, Peus’ article mentions that the composition of breast milk may be beneficial for brain development and thus help prevent depression [69]. Thus, some experts recommend breastfeeding as a source of nutrition for infants up to the age of half a year and believe that breastfeeding is beneficial for lifelong health, including neurological development and mental health [70].

**Figure 2:**
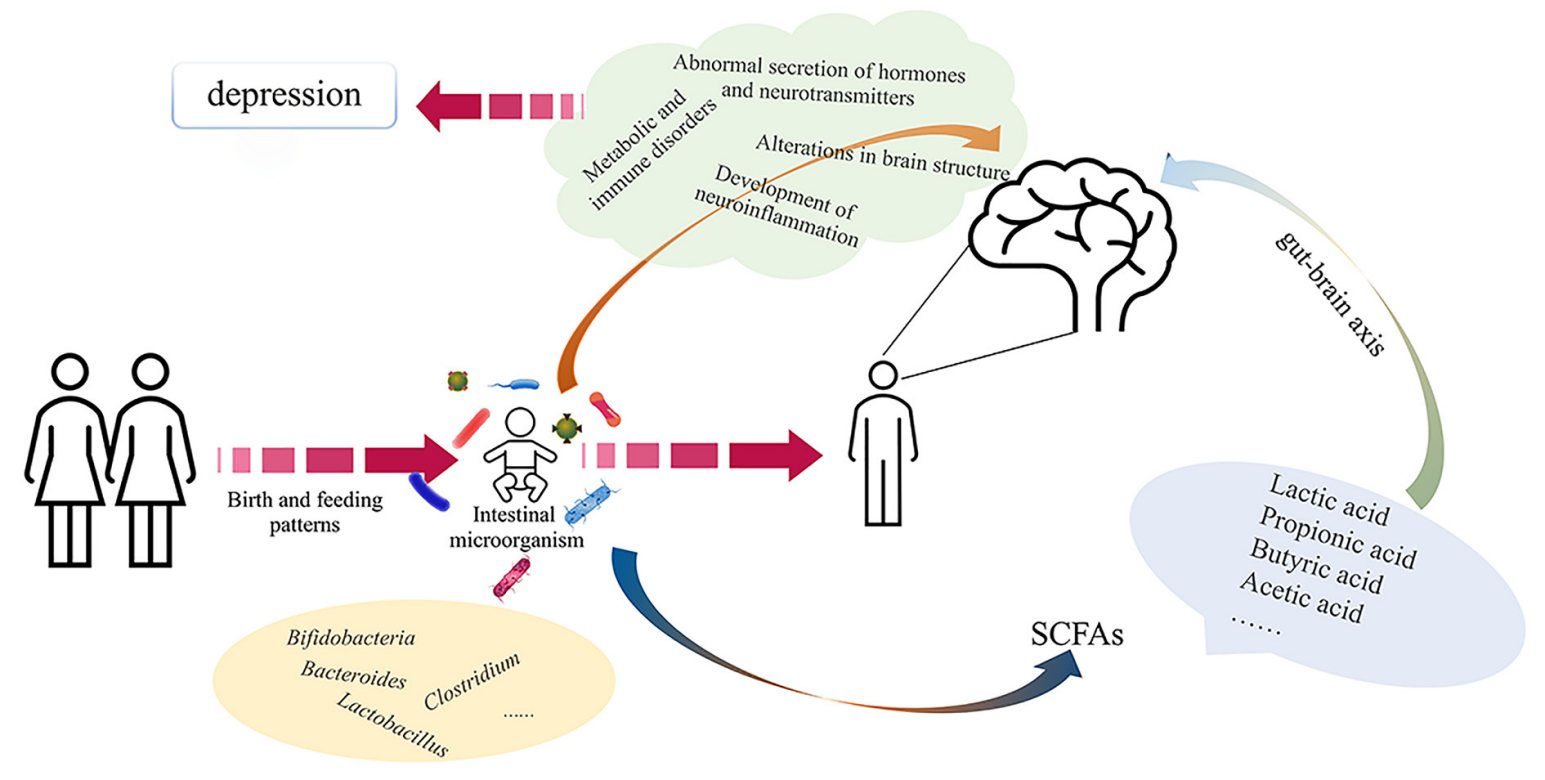
Potential pathways of influence of birth modes and feeding patterns on infants in adulthood

The microbiota affects the formation and regulation of gut–brain axis function [71]. The gut microbiota has a significant impact on processes related to neurotransmitter synthesis and myelination of neurons in the prefrontal cortex and is also involved in the development of the amygdala and hippocampus [72–74]. Gut bacteria are also a source of vitamins, and it is thought that vitamin deficiency is associated with response to antidepressant treatment and may lead to an exacerbation of depressive symptoms [73]. The relationship between gut flora and depression has been frequently reported, and depressive states may lead to alterations in specific gut microbiota species and ultimately to more severe depression [75]. By transplanting feces from depressed patients to rats with depleted microbiota, certain behavioral and physiological features of depressive symptoms can be induced, particularly those related to gut microbiota abundance and diversity, tryptophan metabolism, and immune function [76]. The results of a meta-analysis showed that probiotics such as *Bifidobacterium longum* and *lactobacilli* have a therapeutic effect on depression [77]. Short-chain fatty acids, including acetic acid, propionic acid, butyric acid, and lactic acid, are common metabolites of beneficial bacteria, and the benefits of short-chain fatty acids include reduction of chronic inflammation, prevention of neurological disorders, antioxidant effects, and brain protection through the gut–brain axis. All the above studies illustrate the important role that gut flora plays in depression, so it is very likely that different birth and feeding patterns contribute to the development of mental conditions and neurological disorders in adulthood by influencing the infant’s gut flora, as shown in Figure 2.

### Dietary differences

Food culture has unique features around the world, and the most studied ones are the Mediterranean diet and the Western diet. The Mediterranean diet refers to the diets of Greece, Spain, France, Italy, and other Southern European countries along the Mediterranean coast and is characterized by a diet based on vegetables, fruits, and grains, which is beneficial for human health. The Western diet refers to the diet of the United States, Canada, and some countries in Northern Europe, and this pattern is characterized by a higher intake of meat, sugar, and high-fat foods characterized by a high content of saturated fatty acids, which is detrimental to human health.

In the field of neuroscience, research has concluded that different dietary patterns can have different effects on an individual’s mental state. Studies on the nutritional aspects of depression have reported that the intake of healthy foods is inversely associated with the risk of depression and can even improve depressive symptoms. However, unhealthy Western dietary patterns are associated with an increased risk of depression [78,79]. Several studies have found that the Mediterranean dietary pattern has a protective effect on the brain and reduces the risk of memory loss and antidepressant benefits [80–83]. For example, in adults with depression, a Mediterranean-style diet combined with fish oil supplementation resulted in improved symptoms of depression compared to a control group receiving social support [84]. A cohort study showed that Swedish women’s higher compliance with the Mediterranean diet at midlife was associated with a lower risk of depression in later life [85]. However, Western dietary patterns are strongly associated with mental disorders such as depression and Alzheimer’s disease [86,87]. The results from two cohort studies and one cross-sectional study showed a significant association between a reduced consumption of Western foods and a reduced likelihood of depressive symptoms [88–90].

**Figure 3:**
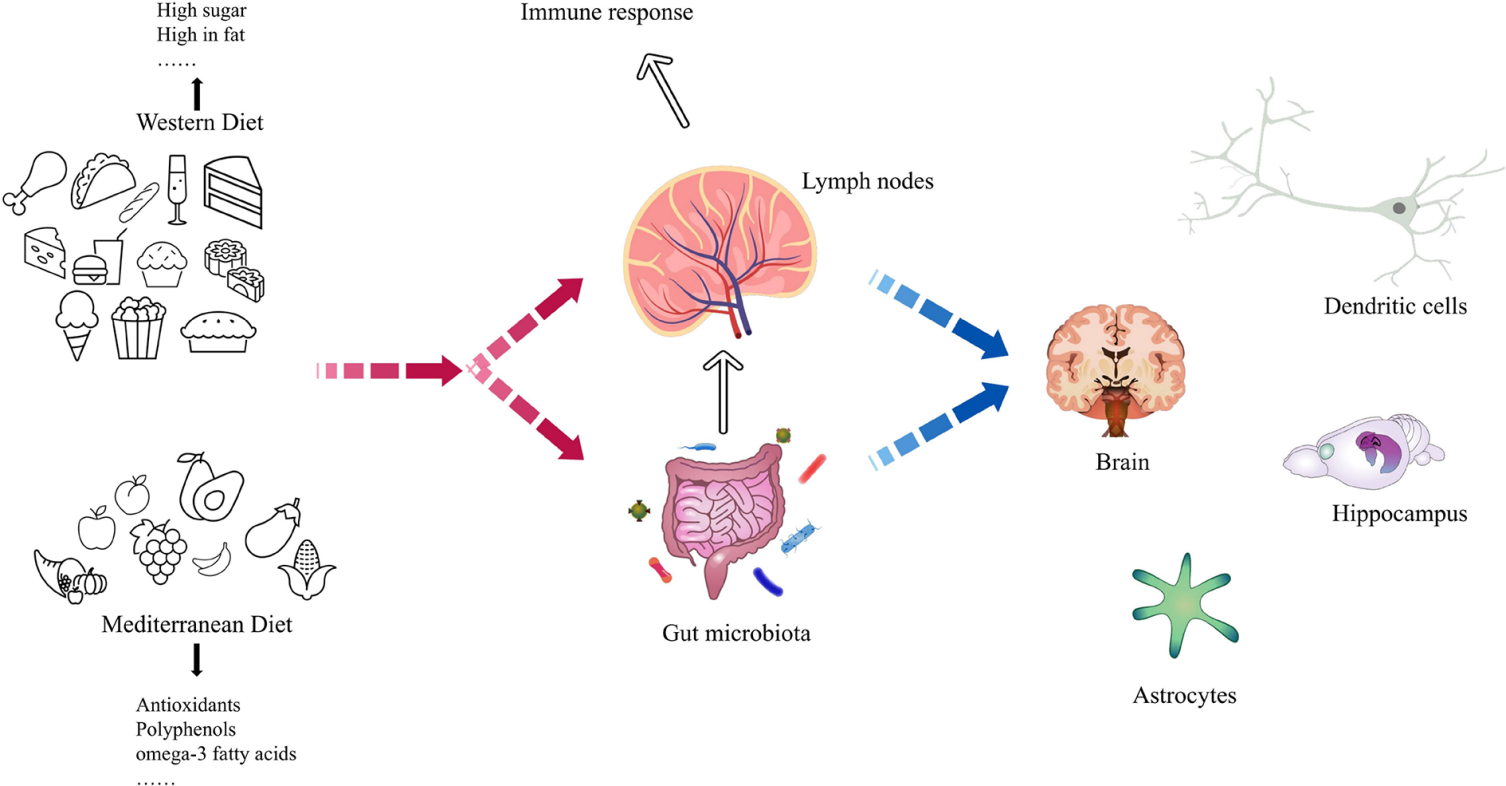
Impact of dietary differences on individuals

In light of these findings, scholars have attempted to explain the effects of diet on the organism in terms of inflammation and gut flora (important influences on depression), among others. Changes in diet can affect the gut microbiota, which may influence mood and involve complex bidirectional interactions between the brain and inflammatory functions and neurotransmitters and neuropeptides [91]. Tran et al. showed that the Western diet may adversely affect the body by disrupting gut microbiota interactions and subsequent innate immune activation, leading to inflammation and metabolic syndrome [92]. Jena found that chronic Western diet intake reduced brain-derived neurotrophic factor levels in the brain and microglia and synaptic plasticity in the hippocampus, while inflammatory signaling was increased in various parts of the mouse body and was accompanied by microglial activation [93]. However, the Mediterranean diet has a positive impact on the immune system and the intestinal microbiota, which helps the body fight against diseases [94]. Studies have shown that fatty acids rich in the Mediterranean diet (e.g., omega-3) have anti-inflammatory and pro-solvency properties in the host, resulting in a protective effect on the organism [95,96]. Thus, the intake of dietary components may act directly on the immune system on the one hand, and on the other hand, it alters the metabolites of the microbiota by influencing the composition and abundance of the intestinal microbiota, which further participates in the immune response of the organism and exerts its protective or harmful effects, as shown in Figure 3.

### Childhood experiences

Psychology reveals that what people do in adulthood reflects their childhood experiences. The environment of childhood shapes one's personality; childhood experiences solidify one’s thought patterns and unconscious inertia. A happy childhood leads to more positive thinking and people can overcome setbacks more quickly even if they encounter them later, whereas unpleasant childhood experiences can place many psychological burdens [97,98]. Research has shown an association between childhood trauma and later adult psychiatric disorders, and this association is particularly pronounced in the context of exposure to bullying, emotional abuse, maltreatment, and parental absence [99]. In addition, people with childhood adversity were more likely to be younger, female, unemployed, single or divorced, have more severe depression and anxiety, have more episodes in their lifetime, be first diagnosed with MDD at a younger age, have more comorbid PTSD, have a poorer quality of life, and have more suicidal thoughts than those with no or less adversity [100]. In contrast, positive childhood experiences play a positive role, and in a study of adult personality disorders, it was found that people with positive childhood experiences recovered more quickly and had a better prognosis than those with adverse childhood experiences [101].

Depression, as a common mental disorder, is also deeply influenced by childhood experiences, and traumatic childhood experiences are thought to be a major risk factor for depression in adulthood [102,103]. In a study exploring the relationship between eight early life stressful events (i.e., sexual abuse, physical abuse, poverty, physical illness/injury, death of a family member, domestic violence, natural disasters, and emotional abuse) and the onset of MDD in adolescents, results showed that individuals who experienced adverse childhood experiences were more likely than normal to develop MDD at age 18 (OR = 2.50; 95% CI: 2.08, 3.00) [104]. Another study showed that psychological abuse and neglect during childhood were most strongly associated with depressive outcomes [105]. This all suggests that children who experienced moderate or high levels of adversity during childhood have a significantly higher risk of depression and severity of depression [106].

Researchers have shown that negative childhood experiences can affect the central nervous system, leading to changes in the expression of stress mediators and neurotransmitters in certain areas of the brain. This, in turn, induces lasting structural and functional changes in the brain, such as in the hippocampus and amygdala [107,108]. A systematic review mentioned that childhood stress or abuse increases hypothalamic‒pituitary‒adrenal axis function and is associated with low hippocampal and anterior cingulate volume, which is strongly associated with the development of MDD in adulthood [109]. Childhood trauma, a risk factor for MDD, has been separately shown to also affect the inflammatory system, as found in a systematic review: patients with MDD who experienced adverse childhood experiences had higher IL-6 levels than MDD-only and healthy control populations [110]. In addition, studies have found that young adults (18–42 years) and middle-aged adults (43–54 years) with a history of physical abuse in childhood exhibit a high allostatic load, which is associated with an increased incidence of depression [102]. To reduce the impact of adverse childhood experiences in later life, some experts suggest that physical activity may mitigate the adverse effects of adverse childhood experiences [111]. Additionally, scholars have suggested the implementation of several interventions to reduce adverse childhood experiences and thus prevent the resulting psychiatric disorders [112].

### Differences in educational level

‘Books are the ladder of human progress’; this means that the progress of human society needs the irrigation of knowledge and that the progress and development of each individual are closely related to education. Education plays an important role in the development of individuals, having a significant impact on both physical and mental health. With gained knowledge and a higher education level, individuals can not only achieve a great sense of psychological comfort and enrichment but also raise their level of thinking. People with different levels of education see the same thing differently. While those with low levels of education may see difficulties and setbacks that are difficult to overcome, those with high levels of education may see these challenges as opportunities. Therefore, the level of education is of great importance to a person's mental health and resilience to stress.

The topic of education level affecting physical and mental health has also been hotly debated by researchers in recent years. Higher levels of education are associated with lower risk of major mental disorders and lower risk of physical illness [113], while there is a significant relationship between low levels of education and self-reported poor mental health [114]. Di Novi et al. investigated the role of higher levels of education not only in protecting people from depression and anxiety symptoms but also in mediating the relationship between physical and mental health shocks and found that among people with serious physical health problems, those with higher levels of education were less likely to experience depression and anxiety symptoms than those with lower levels of education [115]. A study of black populations shows that education is protective against the risk of depression associated with discrimination in men [116]. Another study of a female population in the United States showed that higher education was associated with lower odds of depressive symptoms (more than 12 but less than 16 years, OR 0.70, 95% CI: 0.49–0.98; 16 or more years of education, OR 0.61, 95% CI: 0.40–0.93) [117].

The reasons why the level of education affects physical and mental health may be as follows. (1) The level of education changes people’s perspective and the way they think about problems, and people with a high level of education have a strong ability to resist stress. (2) The level of education affects people’s social status, and people with higher social status tend to have a greater sense of psychological fulfillment. (3) The level of education affects people’s economic level, and people’s economic level and personal happiness are closely related. Given the above research findings and analysis, we support the view that education level affects people’s physical and mental health, and we believe that a high level of education is conducive to the development of people’s mental health and reduces the risk of developing depression.

### Differences in economic level

With the development of society, the economic environment of countries worldwide is improving significantly, and people’s quality of life is improving as well. However, in recent years, due to the spread of the global epidemic, the economic development of many countries and regions has faced serious hindrances, and people from all walks of life have been affected to different degrees. The closure of companies has resulted in many people losing their jobs, and people’s economic incomes have therefore plummeted. The economic level is often closely related to the standard of living and personal happiness. During childhood, the economic level of parents is critical to a child's development. In a review, it was reported that parental economic level is highly influential in determining children’s physical and mental health and future outcomes, including children’s cognitive functions and basic neurobiological parameters that influence brain development [118]. For adults, their economic level plays an important role. In a study on income inequality and the mental health of adults at all levels, the results showed that income inequality at the regional level was associated with poor mental health [119].

The onset and progression of depression are also strongly associated with economic status. A 7-year longitudinal population study showed that a decline in material standard of living was associated with an increase in depressive symptoms and cases of major depression [120]. Similarly, a Korean longitudinal study showed that low economic status was associated with a higher likelihood of depressive symptoms. In particular, the highest likelihood of depressive symptoms was found in the group of children currently at a low level of economic status, indicating the adverse effects of chronic poverty on mental health [121]. Another study showed a correlation between economic hardship caused during the epidemic and episodes of MDD [122]. A significant association between depression and low socioeconomic status was found across all countries in the study by Freeman et al. (*P*≤0.001) [123]. In sum, the economic level plays a crucial role in an individual’s life development. The improvement of economic level enables people to enjoy more favorable material conditions in life, a higher social status, access to greater opportunities, and enhanced ability to resist risks; mentally, people experience a greater sense of fulfillment and happiness, which is beneficial to the development of their physical and mental health, helps reduce the risk of mental disorders, and helps enhance stress resistance.

### Isolation due to epidemic

Since the outbreak of COVID-19, people around the world have been in a state of panic, and the incidence of depression has skyrocketed. There are many reasons behind this phenomenon, and one of the important ones is isolation prevention and control. With the normalization of epidemic prevention and control, controlled social distancing and isolation have become the norm in life. Governments are now quarantining people across regions and in close contact with positive patients for 7–14 days to determine if they are infected with the virus, or even when the epidemic is severe, static management of entire regions for the purpose of quarantine prevention and control. Isolation measures focus on confining people to a small, enclosed space for long periods of time without direct contact or communication with others, which can lead to serious mental health problems.

Several research studies have shown that isolation measures can induce severe depressive symptoms [124,125]. The results of a questionnaire survey of French students in home isolation during the epidemic showed prevalence rates of 11.4%, 22.4%, 24.7%, 16.1%, and 27.5% for suicidal ideation, severe distress, high perceived stress, severe depression, and high anxiety, respectively [126]. A study of the psychological status of people with disabilities during the epidemic showed that higher social isolation was a significant predictor of depression and anxiety symptoms [127]. A Norwegian epidemiological study showed that a state of fear of isolation caused by isolation during the epidemic was associated with specific symptoms of MDD [128]. These findings all suggest that isolation during an epidemic may severely affect people’s mental health and even lead to the onset of depression.

The mechanism behind this is complex, and scientists have tried to determine the answer through animal simulation. When socially isolated rats and normal rats were studied, it was found that socially isolated rats exhibited more pronounced depressive behaviors. Analysis of this mechanism showed that socially isolated rats had significantly lower copper and zinc concentrations and significantly lower catalase activity in the prefrontal cortex and hippocampus, and since the zinc–copper ratio is an important predictor of depressive outcome [129] and, in addition, catalase treatment improves astrocyte function [130], the changes in micronutrient content and reduced catalase activity caused by social isolation may be responsible for the depression-like behavior of these animals [131]. Du Preez et al. found that chronic stress and social isolation promote depressive-like behaviors, alter microglial and astrocyte biology, and reduce hippocampal neurogenesis in male mice [132]. PPAR-α is a newly discovered target involved in emotion-behavior regulation and can ameliorate anxiety and depression-like behavior by modulating neuroinflammation-induced neuroprotective effects and by enhancing neurosteroid biosynthesis following endogenous or synthetic ligand stimulation. Matrisciano et al. found that social isolation stress can induce the brain to undergo PPAR-α downregulation related to epigenetic markers [133]. The above studies suggest that social isolation leads to a disruption of the body’s immune system and that brain regions within the brain, such as the hippocampus, are severely affected, which may be an important way in which social isolation induces depression.

The above-mentioned information on the influence of acquired factors on the onset and progression of depression has been compiled and is shown in Table 2.

**Table 2:** List of acquired factors mediating the development of depression

Acquired factors	Point of view	Source
Birth and feeding patterns influence depression through gut flora	There is a developmental window early in life when perturbations in the gut microbiota can have lasting effects on the development of the central nervous system, which may lead to microglia activation and neuroinflammation in adulthood, which in turn can lead to neurological disorders such as depression.	[59,60]
	Depressive states may lead to alterations in specific gut microbiota species and eventually lead to more severe depression.	[75]
	Certain behavioral and physiological features of depressive symptoms, particularly those related to gut microbiota abundance and diversity, tryptophan metabolism, and immune function, can be induced by transplanting feces from depressed patients into rats with depleted microbiota.	[76]
	Probiotics such as *Bifidobacterium longum* and *lactobacillus* have a therapeutic effect on depression	[77]
	Birth and feeding practices influence the type and abundance of the infant’s gut flora.	[61,62]
	When the baby is delivered by normal birth, the composition of the baby’s flora is similar to that of the mother's vagina; when the baby is delivered by cesarean section, the composition of the baby’s flora is similar to that of the flora on the mother's skin.	[63]
	Breastfed individuals have a higher percentage of beneficial bacteria in their gut flora and are less likely to develop mental illness than nonbreastfed individuals.	[65]
	Beneficial bacterial genera such as *Bifidobacterium*, *Bacteroides*, *Clostridium*, and *Lactobacillus* predominate in breastfed infants.	[66,67]
	The common colonizing flora in infants born by cesarean section are *Enterococcus*, *Enterobacter*, and *Klebsiella*. These pathogenic bacteria usually have adverse effects on the organism.	[64]
	Breastfeeding facilitates resistance to social stress.	[68]
	The components of breast milk may be beneficial for brain development, thus helping to prevent depression.	[69]
	Breastfeeding is beneficial for lifelong health, including neurological development and mental health.	[70]
	Intestinal bacteria are a source of vitamins, which can contribute to depressive symptoms by causing vitamin deficiencies.	[73]
Dietary differences	Unhealthy Western dietary patterns are associated with an increased risk of depression.	[78,79]
	Mediterranean dietary patterns have a protective effect on the brain, reducing the risk of memory loss and antidepressant benefits.	[80–83]
	A Mediterranean-style diet combined with fish oil supplementation resulted in improved symptoms of depression.	[84]
	Higher adherence to a Mediterranean-style diet at midlife is associated with a lower risk of depression in later life.	[85]
	Western dietary patterns are strongly associated with psychiatric disorders, such as depression and Alzheimer’s disease.	[86,87]
	There is a clear association between reduced consumption of Western foods and reduced likelihood of depressive symptoms.	[88–90]
	Dietary changes affect the gut microbiota, which may influence mood and involve complex bidirectional interactions between brain and inflammatory functions and neurotransmitters and neuropeptides.	[91]
	Western diet may adversely affect the body by disrupting gut microbiota interactions and subsequent innate immune activation, leading to inflammation and metabolic syndrome.	[92]
	Chronic Western dietary intake reduces brain-derived neurotrophic factor levels in the brain, as well as microglia and synaptic plasticity in the hippocampus, while increasing inflammatory signaling in various parts of the mouse body, accompanied by microglia activation.	[93]
	Mediterranean diet rich in fatty acids (e.g., omega-3) has anti-inflammatory and pro-soluble effects in the host, resulting in a protective effect on the organism.	[95,96]
Childhood experiences	Traumatic childhood experiences are considered a major risk factor for depression in adulthood, with young and middle-aged adults with a history of physical abuse in childhood exhibiting high allogeneic load, which is associated with an increased incidence of depression.	[102,103]
	People who have experienced adverse childhood experiences are more likely than normal to develop MDD by age 18.	[104]
	Childhood psychological abuse and neglect are most strongly associated with depressive outcomes.	[105]
	Children who experienced moderate or high levels of adversity during childhood had a significantly higher risk of depression and severity of depression.	[106]
	Negative childhood experiences can affect the central nervous system, leading to changes in the expression of stress mediators and neurotransmitters in certain areas of the brain. This, in turn, can induce lasting structural and functional changes in the brain, such as the hippocampus and amygdala.	[107,108]
	Childhood stress or abuse increases the function of the hypothalamic‒pituitary‒adrenal axis and is associated with low hippocampal and anterior cingulate volume, which is strongly associated with the development of MDD in adulthood.	[109]
	Childhood trauma is a risk factor for MDD, and patients with MDD who have experienced adverse childhood experiences have higher IL-6 levels than those with MDD only and healthy controls.	[110]
Differences in educational level	Higher levels of education are associated with a lower risk of major mental disorders and most physical illnesses.	[113]
	There is a significant relationship between low levels of education and self-reported poor mental health.	[114]
	Among individuals with serious physical health problems, those with high levels of education were less likely to have symptoms of depression and anxiety than those with low levels of education.	[115]
	Studies of black populations have shown that education has a protective effect on the risk of depression for men who experience discrimination [107].	[116]
	Studies of female populations in the United States have shown that higher levels of education are associated with lower odds of depressive symptoms.	[117]
Differences in economic level	Parents’ economic level has a strong influence on determining children’s physical and mental health and future outcomes, including children’s cognitive functioning and basic neurobiological parameters that influence brain development.	[118]
	Area-level income inequality is associated with poor mental health in adults.	[119]
	Declining material living standards are associated with increased cases of depressive symptoms and major depression.	[120]
	The likelihood of depressive symptoms is highest among the group of children who are currently in a low economic status, indicating the adverse effects of chronic poverty on mental health.	[121]
	Economic hardship caused during the epidemic has been associated with episodes of MDD.	[122]
	There is a clear association between depression and low socioeconomic status in all countries.	[123]
Isolation due to epidemic	Isolation measures can induce severe depressive symptoms.	[124,125]
	Higher levels of social isolation are a significant predictor of depressive and anxiety symptoms.	[126,127]
	Fear states caused by isolation during epidemics are associated with specific symptoms of MDD.	[128]
	Socially isolated rats had significantly lower copper and zinc concentrations in the prefrontal cortex and hippocampus and significantly lower catalase activity, which may account for the depression-like behavior in these animals.	[131]
	Chronic stress and social isolation promote depression-like behaviors, alter microglia and astrocyte biology, and reduce hippocampal neurogenesis in male mice.	[132]
	PPAR-α enhances neurosteroid biosynthesis to improve anxiety and depression-like behaviors, while social isolation stress induces a down-regulation of PPAR-α in the brain associated with epigenetic markers.	[133]

## Discussion

This review suggests that the onset and development of depression are influenced by a number of factors, which can be attributed to two aspects, congenital and acquired factors, and that these two interact and complement each other. Among the congenital factors, genetic mutations and genetic polymorphisms have been shown to be very important for their involvement in the onset and development of depression; from an epigenetic point of view, DNA methylation and histone modifications play a pivotal role. Abnormal genetic changes and epigenetic events identified based on innate factors may become important targets for treatment in subsequent studies of depression, such as the development of antidepressant drugs, and may even become useful new therapies. In the study of acquired factors, birth and feeding patterns, dietary habits, childhood experiences, economic and educational levels, and isolation during the epidemic were taken into account. Synthesizing the literature, it was found that normal birth and breastfeeding facilitated the regulation of the infant’s intestinal flora, increasing its proportion of beneficial bacteria and strengthening the infant’s resistance, which may reduce its risk of depression. In the study of dietary patterns, it was found that the Mediterranean dietary pattern was beneficial, while the Western dietary pattern increased the risk of illness in the organism, which may be associated with intestinal flora and inflammation. Good childhood experiences showed a positive association with mental health in adulthood, while poor childhood experiences significantly increased the risk of depressive disorders in adulthood. Good economic and educational levels gave people a better sense of well-being and satisfaction, and the risk of depression decreased, and vice versa. Isolation during the epidemic led to a sense of isolation, which may induce severe depressive disorders.

Although the above evidence may demonstrate that both congenital and acquired factors play an important role in the onset and development of depression, there are certain limitations to this article: (1) the current diagnostic criteria for depression are not very consistent across countries worldwide, which may lead to a certain degree of heterogeneity in research findings worldwide. (2) Because of individual differences, it is not certain that each individual patient will be affected consistently, and the same factor will have different effects on different individuals, so specific analysis is needed for specific problems. (3) Since the organism is a holistic and complex regulatory network, the interaction of various factors on the organism is complex and high-dimensional; therefore, more research is needed in this field to deeply explore the specific mechanisms behind this.

In summary, the above findings suggest that multiple congenital and acquired factors affect individuals’ mental health and resilience in different ways, which have important implications for the onset and development of depression. This also gives us some insights: (1) Research on depression can be analyzed in depth in terms of gene mutations, gene polymorphisms and epigenetics. (2) To avoid the occurrence of depression in offspring as much as possible, parents should try to control the root cause as much as possible, such as choosing normal birth and breastfeeding in the birth and feeding patterns of their children, choosing healthy dietary patterns from an early age, providing support for their children during their growth, and creating a good living environment for their children during their growth process. Depression as a mental illness has seriously threatened people's physical and mental health, and only a more in-depth and thorough study of depression can effectively promote the prevention and treatment of this illness. We hope that after in-depth exploration of this field, we can effectively reduce the occurrence of depression in clinical practice and inhibit the process of depression development.

## Conclusion

In studying the factors affecting depression, the study of genetic factors revealed that genetic mutations, genetic polymorphisms, and epigenetic inheritance play an important role; by analyzing acquired factors, including birth and feeding practices, dietary patterns, childhood experiences, education level, economic level, and isolation and prevention during epidemics, we found that differences in living standards and living environments also have a significant impact on individual depression. Through comprehensive analysis and discussion, we found that some innate and acquired factors can act on the organism's immune system and gut flora, which in turn can lead to susceptibility to depression. We hope that this discussion will contribute to the development of depression research and help in the clinical management of depressed patients.

## Abbreviations

ACTH: Adreno-cortico-tropic-hormone
BDNF: brain-derived neurotrophic factor
CRH: Corticotropin-releasing hormone
DNMT: DNA methyltransferase
GDNF: glial cell-derived neurotrophic factor
MDD: major depressive disorder
PTSD: Post-Traumatic Stress Disorder
